# Comparison of the effects of clear aligners and fixed appliances on the oral microbiota and reactive oxygen species: a prospective study

**DOI:** 10.3389/fcimb.2025.1738047

**Published:** 2026-01-23

**Authors:** Min Xu, Guiding Li, Jingjun Tian, Feifei Xie, Jingpeng Zuo, Jiangtian Hu, Kang Yin, Wei Wang

**Affiliations:** 1 Yunnan Key Laboratory of Stomatology & Department of Orthodontics, The Affiliated Stomatology Hospital, Kunming Medical University, Kunming, China; 2 Department of Orthodontics, Stomatology Hospital of Yunnan Province, Kunming, China; 3 Yunnan Key Laboratory of Stomatology, The Affiliated Stomatology Hospital, Kunming Medical University, Kunming, China

**Keywords:** 16s rDNA sequencing, clear aligners, fixed appliances, oral microbiota, reactive oxygen species

## Abstract

**Objective:**

This study aimed to compare the effects of clear aligners (CA) and fixed appliances (FA) on periodontal indices, oral microbiota, and oxidative stress markers. Potential associations between microbial changes, oxidative stress, and periodontal health were also explored.

**Methods:**

Twenty-four orthodontic patients matched at baseline were randomly allocated to the CA (n = 12) and FA (n = 12) groups. Saliva, supragingival plaque, and gingival crevicular fluid (GCF) were collected at baseline (T0), 3 months (T1), and 6 months (T2). Periodontal indices, including plaque index (PI), gingival index (GI), probing depth (PD), bleeding on probing (BOP) were recorded. Microbial composition was assessed via 16S rDNA sequencing. 8-Hydroxy-2’-deoxyguanosine (8-OHdG, a stable biomarker of oxidative DNA damage reflecting ROS levels) in saliva and GCF was quantified using double-antibody sandwich ELISA. Associations were analyzed using Spearman correlation.

**Results:**

PI was significantly higher in the FA group than CA group at T1 (*P* < 0.01) and T2 (*P* < 0.05). BOP was higher in the FA group than CA group at T2 (*P* < 0.05). Pathogenic genera (*Prevotella*, *Veillonella*) were enriched in the FA group, while health-associated *Rothia* and *Lautropia* predominated in the CA group (*P* < 0.05). In GCF, 8-OHdG levels were higher in the FA group than CA group at T2 (*P* < 0.001). In saliva, *Prevotella* positively correlated with 8-OHdG in the FA group (*r* = 0.61, *P* < 0.05); *Prevotella* negatively correlated with *Rothia* in both groups (*r* = -0.90, *P* < 0.001).

**Conclusions:**

Fixed appliances were associated with enrichment of pathogenic taxa, elevated oxidative stress markers, and worse periodontal indices, potentially linked to higher periodontitis risk. Clear aligners showed less microbial disruption and health-associated taxa enrichment. The *Prevotella*-8-OHdG-*Rothia* axis highlights microbiota and oxidative stress interactions as promising targets for preventing orthodontic treatment related periodontal complications.

## 1 Introduction

Fixed appliances are the most widely used devices in clinical orthodontics. They consist of bands, brackets, and archwires, providing broad indications with precise control of tooth movement (Fleming and Johal, 2010). These appliances are bonded directly to the enamel surface, which easily create plaque-retentive areas that hinder oral hygiene. This increases the risks of plaque accumulation, which can lead to enamel demineralization, dental caries, and gingival inflammation (Ren et al., 2014; Miller et al., 2016; Salerno et al., 2024). In contrast, clear aligners are removable appliances which were manufactured from thermoplastic materials using advanced computer-aided design and computer-aided manufacturing (CAD/CAM) technology with characteristic of esthetic appearance and comfort (Tartaglia et al., 2021; Bichu et al., 2023). Patients can easily remove clear aligners to clean their teeth. This helps reduce plaque retention and maintains periodontal health. However, effective treatment requires wearing aligners for more than 22 hours daily, which may compromise the natural cleansing effect of saliva and promote plaque accumulation on tooth surfaces and inside the aligners (Galan-Lopez et al., 2019). Therefore, a systematic comparison of these two orthodontic appliances and their impact on the oral microenvironment is essential for optimizing treatment strategies.

Recent studies have mainly compared the effects of fixed appliances and clear aligners on clinical periodontal parameters such as plaque index and bleeding on probing, with a prevailing consensus that clear aligners exert less adverse impact on periodontal health (Chhibber et al., 2018; Jiang et al., 2018; Llera-Romero et al., 2023). Advances in molecular biology have enabled the use of high-throughput sequencing, such as 16S rRNA gene sequencing, to analyze changes in the oral microbiota. These methods sequence conserved bacterial gene regions in environmental samples, providing a comprehensive profile of microbial composition, diversity, and community structure. They also offer high sensitivity, strong specificity, and rapid detection (Youssef et al., 2009; Overmyer et al., 2021). Despite these advances, the influence of different orthodontic appliances on oral microbiome homeostasis remains debated. Some studies have shown lower subgingival microbial abundance in clear aligners users compared with fixed appliance patients, suggesting a greater capacity for maintaining microbial balance (Levrini et al., 2015). Other reports have indicated that fixed appliances may promote colonization by cariogenic bacteria and shift microbial metabolism toward an acidogenic phenotype, whereas clear aligners appear to trigger only transient inflammatory responses (Gong et al., 2024). Conversely, Wang Q et al. have found through metagenomic analysis of saliva that both orthodontic methods exert microbial dysbiosis, and clear aligners show no significant advantage (Wang et al., 2019). These inconsistencies highlight the need for further studies integrating multidimensional indicators to clarify the underlying mechanisms.

Reactive oxygen species (ROS), a collective term for oxygen-derived reactive molecules such as hydroxyl radicals and superoxide anions, play extensive biological roles in living organisms (Dumitru et al., 2025). The sources of ROS can be classified into two major categories: endogenous and exogenous. Endogenous sources derive from cellular and mitochondrial metabolism, immune activity, and oral microbiome imbalance. While, exogenous sources originate from alcohol, smoking, xenobiotics, dental materials, diets, and radiation (Chen et al., 2019). Previous study showed that ROS play dual roles in maintaining oral microbial homeostasis (Baltacıoğlu et al., 2014). During dysbiosis, pathogenic bacteria, including *Fusobacterium nucleatum* and *Porphyromonas gingivalis*, stimulate host neutrophils to release excessive ROS, thereby inducing oxidative stress and driving chronic inflammation. On the other side, physiologically generated ROS from commensal bacteria can suppress pathogen colonization and support microbial balance (Tamaki et al., 2009; Kang et al., 2019; Sies and Jones, 2020). Substantial evidence has linked ROS to the pathogenesis of periodontitis and dental caries (Liu et al., 2017; Yu et al., 2023; Pouliou and Piperi, 2024). However, the dynamic fluctuations of ROS during orthodontic treatment and their underlying interactions with the oral microbiota remain poorly investigated.

Therefore, this study aims to integrates clinical periodontal assessment, 16S rDNA gene sequencing, and ROS detection. It systematically compared the effects of fixed appliances and clear aligners on periodontal indices, oral microbial community structure, and ROS levels. The study further investigates the correlations between ROS, oral microbiota, and clinical periodontal parameters, thereby providing a theoretical basis for the development of antioxidant and antimicrobial materials tailored for orthodontic patients.

## 2 Materials and methods

### 2.1 Ethical approval

This study was approved by the Medical Ethics Committee of the Affiliated Stomatology Hospital, Kunming Medical University (Approval No. KYKQ2024MEC0036; Feb. 2024). Written informed consent was obtained from all adult participants. For participants under 18 years of age, written informed consent was obtained from their legal guardians.

### 2.2 Participant recruitment

A total of 24 patients (10 male and 14 female) were recruited from the Department of Orthodontics of, Affiliated Stomatology Hospital, Kunming Medical University (Kunming, China) from September 2023 to October 2024. The inclusion criteria were as follows: (1) no use of antibiotics, hormone or radiation therapy within three months prior to sampling; (2) non-smoker and no alcohol use; (3) absence of systemic disease; (4) no dental fillings, restorations, and implants; (5) absence of clinically detectable enamel demineralization, dental caries, and periodontitis. The exclusion criteria encompassed preexisting gingivitis, severe periodontal diseases, generalized caries, systemic diseases, pregnancy, and irregular attendance. Eligible participants were randomly allocated to the clear aligners group (CA, n = 12) or fixed appliances group (FA, n = 12). Specifically, the participants in CA group were treated with clear aligners (Invisalign^®^, Align Technology, Calif) and instructed to wear aligners at least 22 hours per day and switch to a new aligner every 10 days. The participants in FA group underwent treatment with self-ligating fixed appliances and nickel-titanium archwires (Damon Q, Ormco, Calif).

All participants were required to answer a questionnaire including 20 questions about their general health, oral hygiene, dietary habits, and wear-time of the aligners at each followup visit monthly during the entire treatment (**Supplementary Table 1**). In addition, the 10-item Perceived Stress Scale (PSS-10) was completed for measuring the participants’ psychological stress at pre-treatment (T0), 3 months (T1), and 6 months (T2) (Choi, 2020). All participants received oral hygiene instructions including standardized Bass tooth brushing method three times daily and use of floss from the same orthodontist (MX).

### 2.3 Sample collection and clinical examination

**Figure 1:**
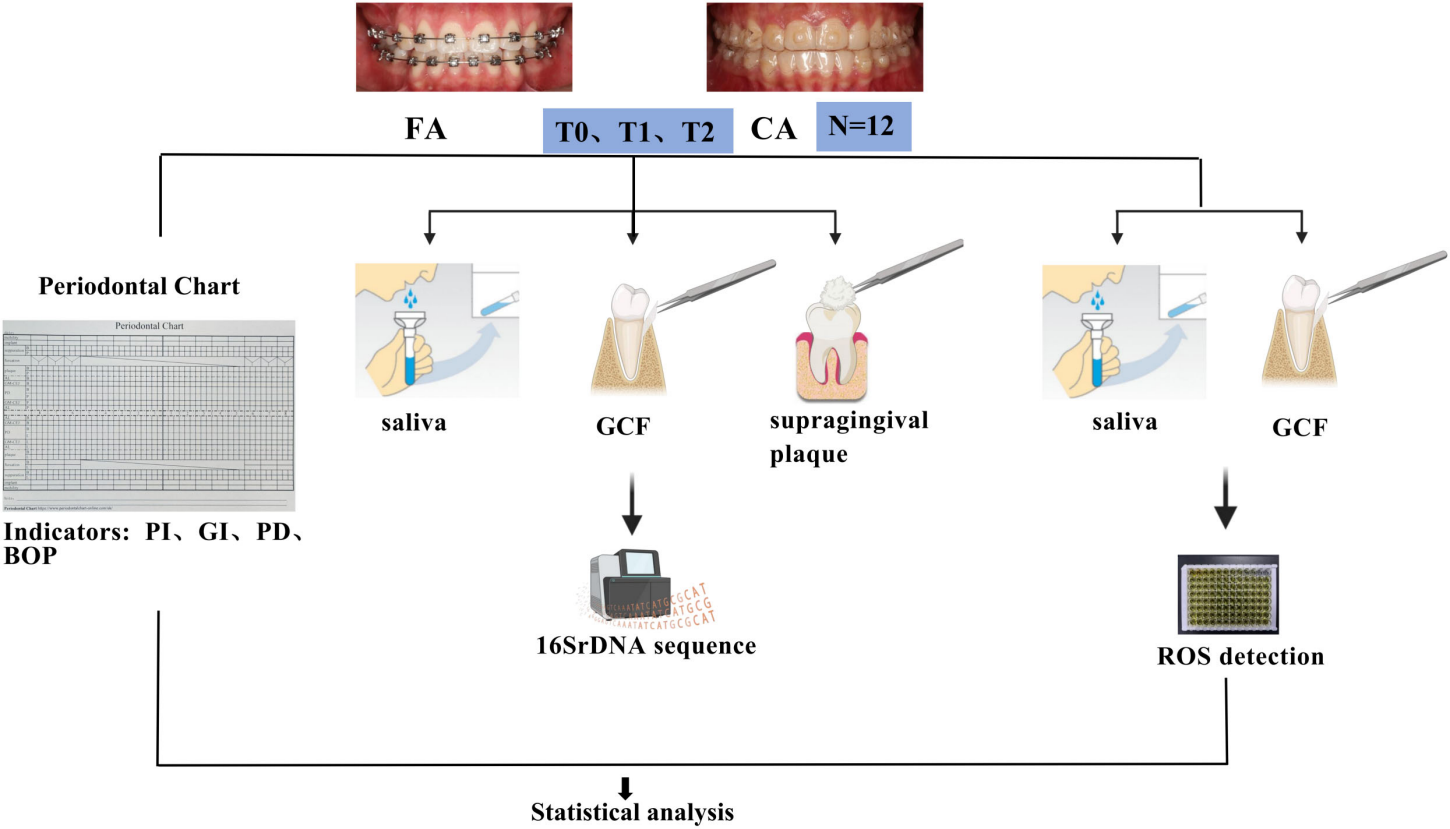
Schematic workflow of the study.

Samples and clinical indices were collected at three time points: baseline (T0), 3 months (T1), and 6 months (T2) after treatment initiation. Specifically, unstimulated whole saliva, supragingival plaque, and gingival crevicular fluid (GCF) were obtained at each time point. For microbial sequencing, saliva, and GCF samples were placed into cryotubes containing TE buffer. For ROS detection, saliva and GCF samples were stored in cryotubes containing PBS buffer. All samples were immediately flash-frozen in liquid nitrogen and subsequently stored at -80 °C until analysis. Prior to each visit, participants were instructed to refrain from food or drink intake for at least 1 hour before saliva collection. Subsequently, following sample collection, periodontal parameters, including probing depth (PD), plaque index (PI), gingival index (GI), and bleeding on probing (BOP), were also recorded. Each measurement was performed in triplicate, and the mean value was used for analysis. The overall study workflow was illustrated in **Figure 1**.

### 2.4 DNA extraction and 16S rDNA gene sequencing

The total microbial DNA was obtained through the Bacterial Genomic DNA Extraction Kit (AU46111-96, BioTeke, China) according to the manufacturer’s instruction manual. The DNA was quantified by Qubit (Invitrogen, USA). Total DNA was amplified by PCR using the universal primer 341F (5’-CCTACGGGNGGCWGCAG-3’) and 805R (5’-GACTACHVGGGTATCTAATCC-3’). The PCR amplification conditions were pre-denaturation at 98°C for 30 s, denaturation at 98°C for 10 s, annealing at 54°C for 30 s, extension at 72 °C for 45 s and 32 cycles. The final extension was at 72 °C for 10 min. The PCR product was purifed using AMPure XT Beads (Beckman Coulter Genomics, Danvers, MA, USA) and quantifed using Qubit (Invitrogen, USA). Qualifed PCR products were evaluated using an Agilent 2100 Bioanalyzer (Agilent, USA) and Illumina library quantitative kits (Kapa Biosciences, Woburn, MA, USA), which were further pooled together and sequenced on an Illumina NovaSeq 6000 (PE250). Sequencing primer were removed from de-multiplexed raw sequences using cutadapt (v1.9). Then, Pairedend reads were merged using FLASH (v1.2.8). The low-quality reads (quality scores<20), short reads (<100bp), and reads containing more than 5% “N” records were trimmed by using the sliding-window algorithm method in fqtrim (v0.94). Quality fltering was performed to obtain high-quality clean tags according to fqtrim. Chimeric sequences were fltered using Vsearch software (v2.3.4). DADA2 was applied for denoising and generating amplicon sequence variants (ASVs). The sequence alignment of species annotation was performed by QIIME2 plugin feature-classifier, and the alignment database was SILVA and NT-16S. Alpha and beta diversities were calculated using QIIME2, Relative abundance was used in bacteria taxonomy.

### 2.5 ROS detection

8-Hydroxy-deoxyguanosine (8-OHdG) is recognized as the most stable product of ROS-mediated DNA damage which can be used as a biomarker of oxidative stress (Öngöz Dede et al., 2013, 2016). Thus, in our study, 8-OHdG was detected to indirectly reflect ROS levels. The level of human 8-OHdG in the saliva and GCF was measured using a human ROS ELISA kit (96T; Meimian, China) based on the double-antibody sandwich method to detect the ROS levels as previously reported (Nishi, 2025). Standards and samples (50 µL) were added to antibody-precoated wells and incubated at 37°C for 30 min, followed by washing with PBS-T (five times). Subsequently, 50 µL of HRP-conjugated antibody was added and incubated at 37°C for 30 min. Color development was achieved by adding 50 µL each of TMB substrate solutions A and B, followed by incubation at 37°C for 10 min in the dark. The reaction was terminated with 50 µL of 2 M H_2_SO_4_, and absorbance was recorded at 450 nm using a microplate reader (SpectraMax^®^ i3x; Molecular Devices, USA). 8-OHdG concentrations were calculated based on a standard curve ranging from 0 to 80 ng/mL (R² > 0.99).

### 2.6 Statistical analysis

Participant characteristics were compared using the independent samples t-test for age and the Chi-square test for gender distribution. Alpha diversity was evaluated using Chao1, ACE, Shannon, and Simpson indices. Differences in relative abundance at the genus level were assessed with the Wilcoxon signed-rank test. Beta diversity was analyzed based on Bray-Curtis distances and visualized through principal coordinates analysis (PCoA). The contribution of individual taxa to intergroup differences was determined using LEfSe analysis (LDA score > 3; Kruskal-Wallis test, *P* < 0.05). Fisher’s exact test was applied to non-replicated categorical data. For two-group comparisons with biological replicates, Mann-Whitney U test was used, and for multiple-group comparisons with replicates, the Kruskal-Wallis test applied. Longitudinal-comparisons of periodontal clinical indices and 8-OHdG levels were performed using linear mixed-effects models, with time and group as fixed effects, and participants as a random effect to account for repeated measures. Correlations between 8-OHdG levels, microbial taxa, and periodontal indices were assessed using Spearman correlation. Statistical significance was set at *P < 0.05, **P < 0.01, ***P < 0.001. All statistical analyses were performed using GraphPad Prism (v9.1.0; GraphPad Software, Inc, San Diego, Calif).

## 3 Results

### 3.1 Characteristics of the study participants

A total of 24 participants were enrolled and allocated into the CA group (clear aligners, n = 12; mean age: 26.17 ± 7.08 years; 9 females, 3 males) and the FA group (Damon Q metal brackets, n = 12; mean age: 20.17 ± 4.06 years; 5 females, 7 males). No significant differences were observed between the two groups with respect to age or sex distribution (*P* > 0.05; **Table 1**).

**Table 1:** Demographic characteristics of the study participants.

Group	Sex (n)		Age (years, mean ± SD)
	Male	Female	
CA	3	9	26.17 ± 7.08
FA	7	5	20.17 ± 4.06

The frequency of tooth brushing, flossing using and desserts consumption were reported at T0, T1, T2 as shown in **Table 2**. No significant differences were found in FA group and CA group at any time points (T0, T1, T2) (*P* > 0.05).

**Table 2:** Tooth brushing frequency at different time points (mean ± SD, times/day).

Group	FA			CA		
	T0	T1	T2	T0	T1	T2
Brushing	1.92 ± 0.29	2.83 ± 0.55*	3.00 ± 0.43*	1.92 ± 0.29	2.83 ± 0.39*	2.92 ± 0.51*
Flossing	0.33 ± 0.49	2.33 ± 0.49	2.17 ± 0.58	0.42 ± 0.51	2.42 ± 0.51	2.25 ± 0.45
Dessert	2.58 ± 0.51	1.25 ± 0.62	1.33 ± 0.49	2.42 ± 0.67	1.17 ± 0.58	1.25 ± 0.62

Values are presented as mean ± standard deviation. Brushing: How many times does a patient brush their teeth daily (in FA group, T1 vs T0, *P* < 0.05; T2 vs T0, *P* < 0.05. in CA group, T1 vs T0, *P* < 0.05; T2 vs T0, *P* < 0.05). Flossing: How many times does a patient floss daily. Dessert: How many times does a patient eat dessert weekly.

PSS-10 items were used to measure psychological stress in order to mitigate the confounding factor which might affect the ROS detection. The PSS-10 is a 10-item questionnaire with each item scored on a 5-point Likert scale (0 = never, 4 = very often). Descriptive statistics for the PSS-10 items are presented in **Supplementary Table 2**. No significant differences between FA group and CA group were found (*P* > 0.05) at T0, T1, and T2 as shown in **Supplementary Table 2**. Similarly, there were no significant differences between T0, T1, and T2 whether in FA group or CA group in the treatment duration (**Supplementary Table 2**).

### 3.2 Clinical periodontal indices

**Figure 2:**
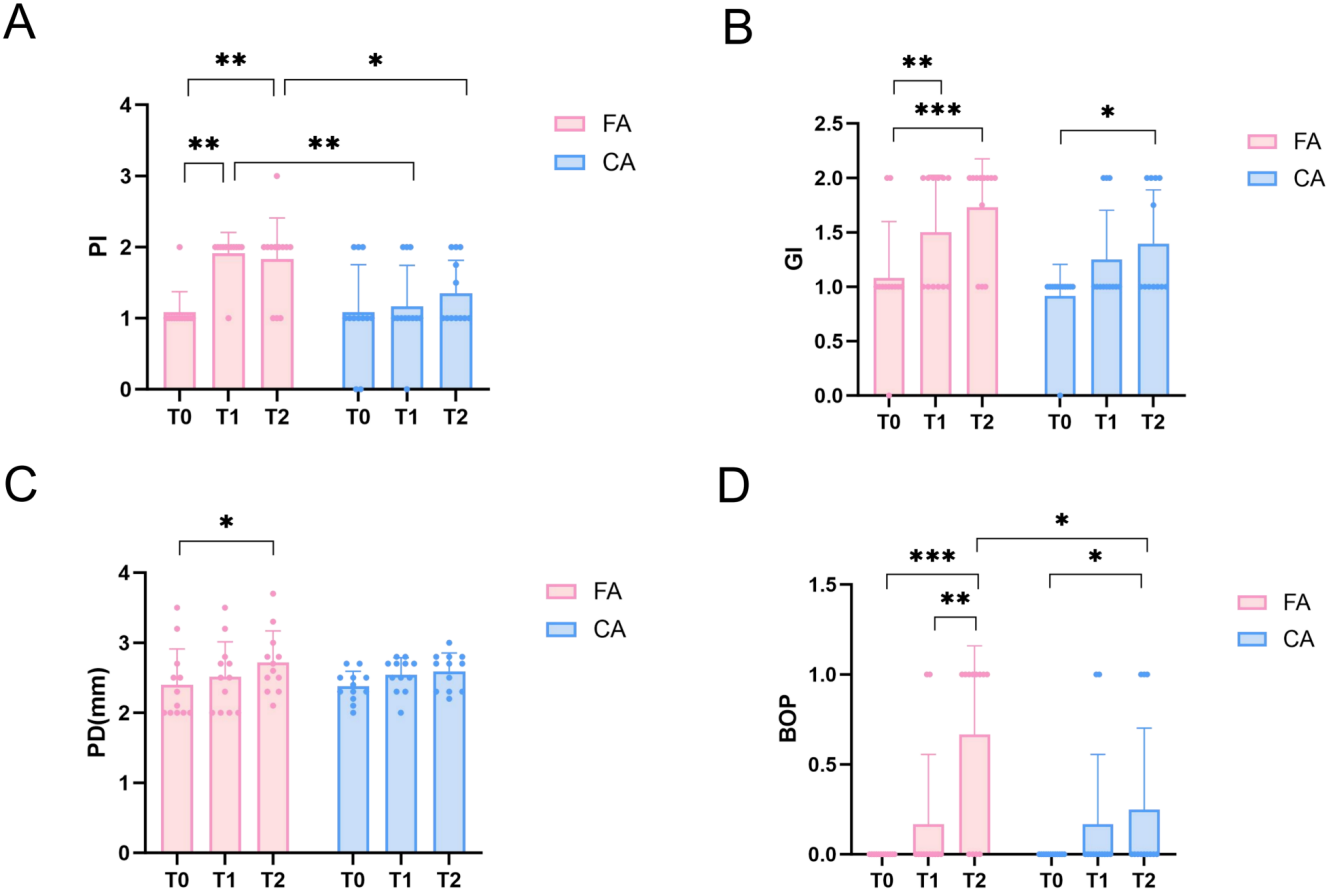
Clinical periodontal indices in the FA and CA groups at T0, T1, and T2. **(A)** Plaque index (PI); **(B)** Gingival index (GI); **(C)** Probing depth (PD); **(D)** Bleeding on probing (BOP). Data are presented as mean ± SD. *P < 0.05, **P < 0.01, ***P < 0.001.

Distinct trends were observed in the clinical periodontal indices between the CA and FA group (**Figure 2**). As shown in **Figure 2A**, PI in FA group increased significantly at T1 and T2 compared with T0 (*P* < 0.01). Additionally, PI in FA group at T1 and T2 were also significantly higher than CA group (T1: *P* < 0.01, T2: *P* < 0.05). As shown in **Figure 2B**, GI in FA group showed a significant increase at T1 and T2 compared with T0 (T0 vs T1, *P* < 0.01, T0 vs T2, *P* < 0.001). GI in CA group also significantly increase at T2 compared with T0 (*P* < 0.05). PD in the FA group showed a significant increase at T2 compared with T0 (*P* < 0.05) as shown in **Figure 2C**. No significant differences of PD were found in CA group (*P* > 0.05). BOP in FA group showed a significant increase at T2 compared with T0 and T1 (T2 vs T0, *P* < 0.001, T2 vs T1, *P* < 0.01) as shown in **Figure 2D**. Similarly, BOP in the CA group also showed a significant increase at T2 compared with T0 (*P* < 0.05). Moreover, BOP was significantly higher in the FA group than CA group at T2 (*P* < 0.05).

### 3.3 Sequencing data and quality control

To investigate composition changes of the microbial communities in salivary, supragingival plaque, and GCF, all samples were sequenced with Illumina MiSeq technology. A total of 14, 459, 037 valid reads were obtained from the 12 subjects, averaging 72, 295 reads per sample, and the majority (>99.9%) of reads spanned 400–500 bp (**Supplementary Figure 1**). Based on the obtained ASV abundance table, a Venn diagram was used to intuitively display the number of ASVs that are shared among and unique to each group (**Supplementary Figure 2**).

### 3.4 Microbiome diversity and richness

#### 3.4.1 Alpha-diversity analysis

**Figure 3:**
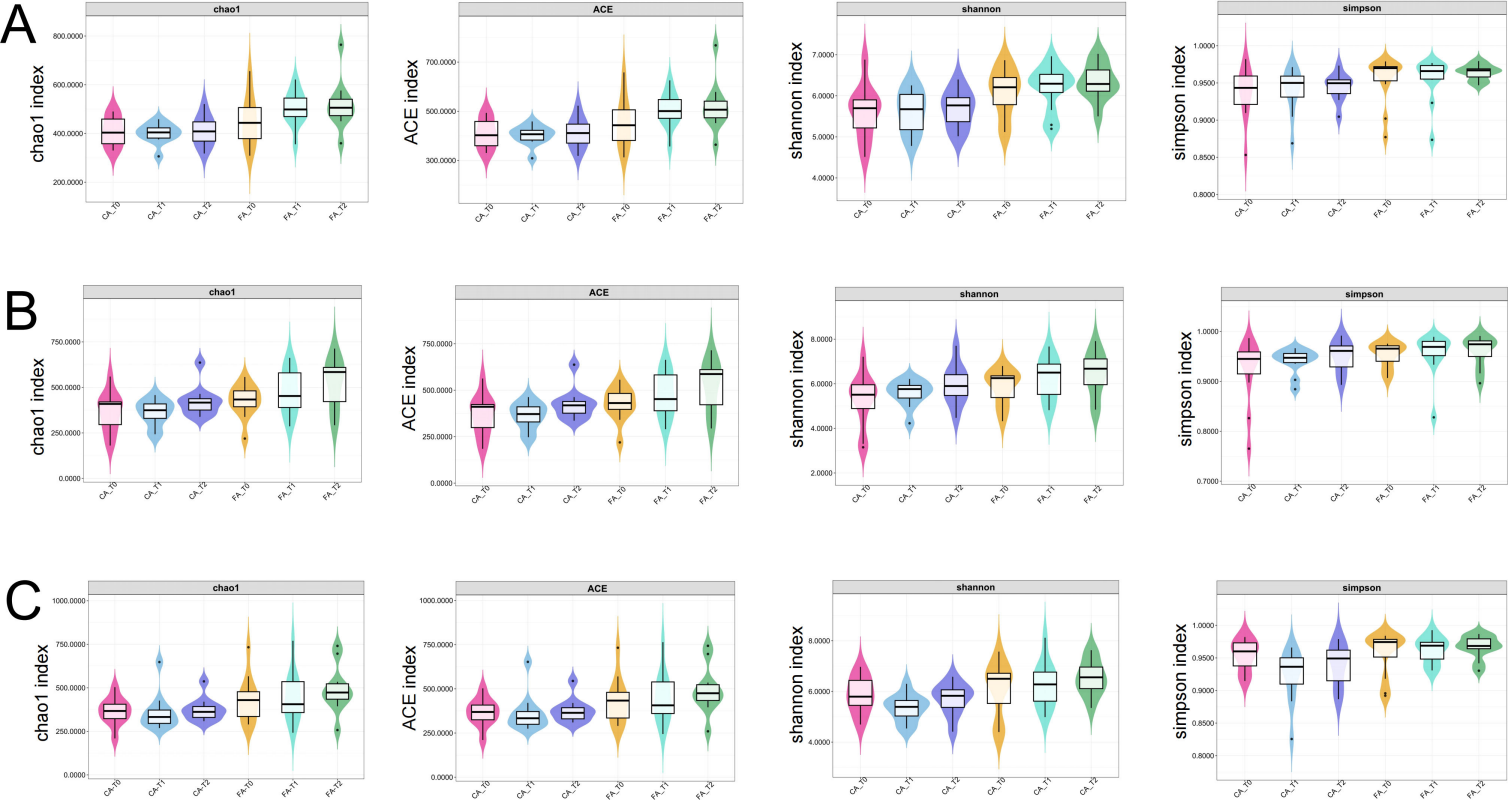
Alpha diversity of the oral microbiota in FA and CA groups. **(A)** Saliva samples (Chao1, ACE, Shannon, and Simpson indices); **(B)** Supragingival plaque samples (Chao1, ACE, Shannon, and Simpson indices); **(C)** GCF (Chao1, ACE, Shannon, and Simpson indices).

Alpha-diversity was evaluated at the species level using amplicon sequence variants (ASVs), with richness estimated by the Chao1 and ACE indices and evenness assessed by the Shannon and Simpson indices. In saliva samples (**Figure 3A**), FA group showed an upward trend in richness (Chao1 and ACE) from T0 to T2, whereas evenness (Shannon and Simpson) remained stable. In CA group, all indices were stable across time points. No significant differences were observed within groups over time or between groups at any time point (*P* > 0.05).

In supragingival plaque samples (**Figure 3B**), the richness indices and evenness indices in FA group increased gradually throughout treatment, although these changes were not statistically significant (*P* > 0.05). In CA group, richness indices (Chao1 and ACE) decreased slightly at T1 and recovered at T2, while evenness indices (Shannon and Simpson) increased progressively, though none of these changes were significant (*P* > 0.05).

In GCF samples (**Figure 3C**), both groups displayed a slight reduction in all indices at T1, followed by an increase at T2, but no significant differences were detected (*P* > 0.05).

#### 3.4.2 Beta-diversity analysis

**Figure 4:**
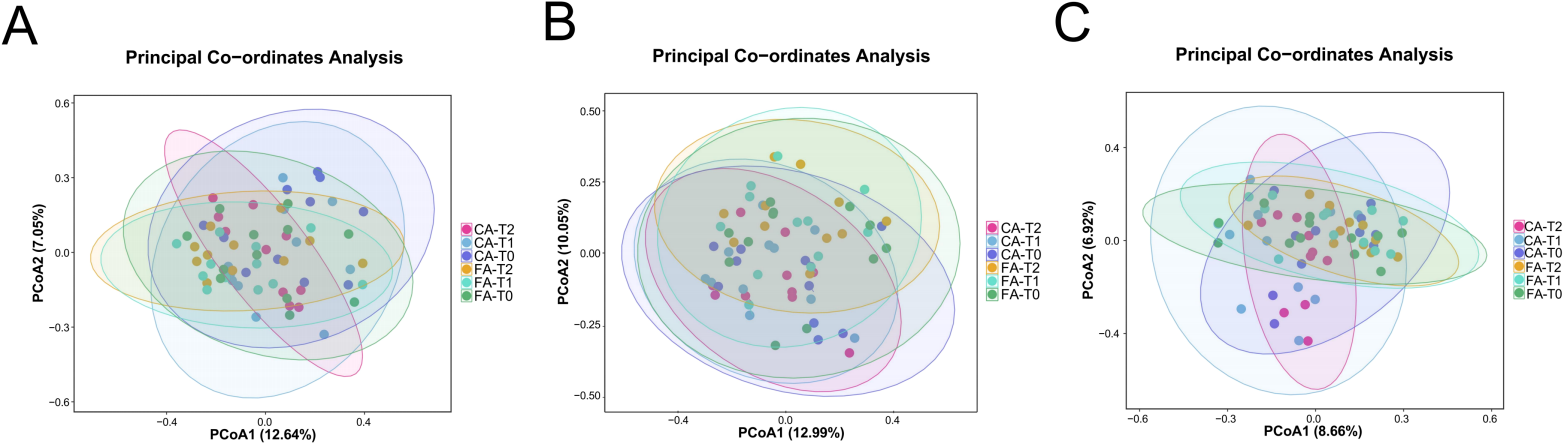
Beta diversity of the oral microbiota in FA and CA groups. **(A)** Saliva; **(B)** Supragingival plaque; **(C)** GCF.

Beta diversity was evaluated using PCoA based on Bray-Curtis distances. In saliva and supragingival plaque, distinct separation trends were observed between the FA and CA group (**Figures 4A, B**). However, no separation trends were observed between the FA and CA group in GCF (**Figure 4C**). Ellipses in the plots represent the 95% confidence intervals, which illustrate the clustering patterns of samples within each group.

### 3.5 Microbial community composition and differential analysis

**Figure 5:**
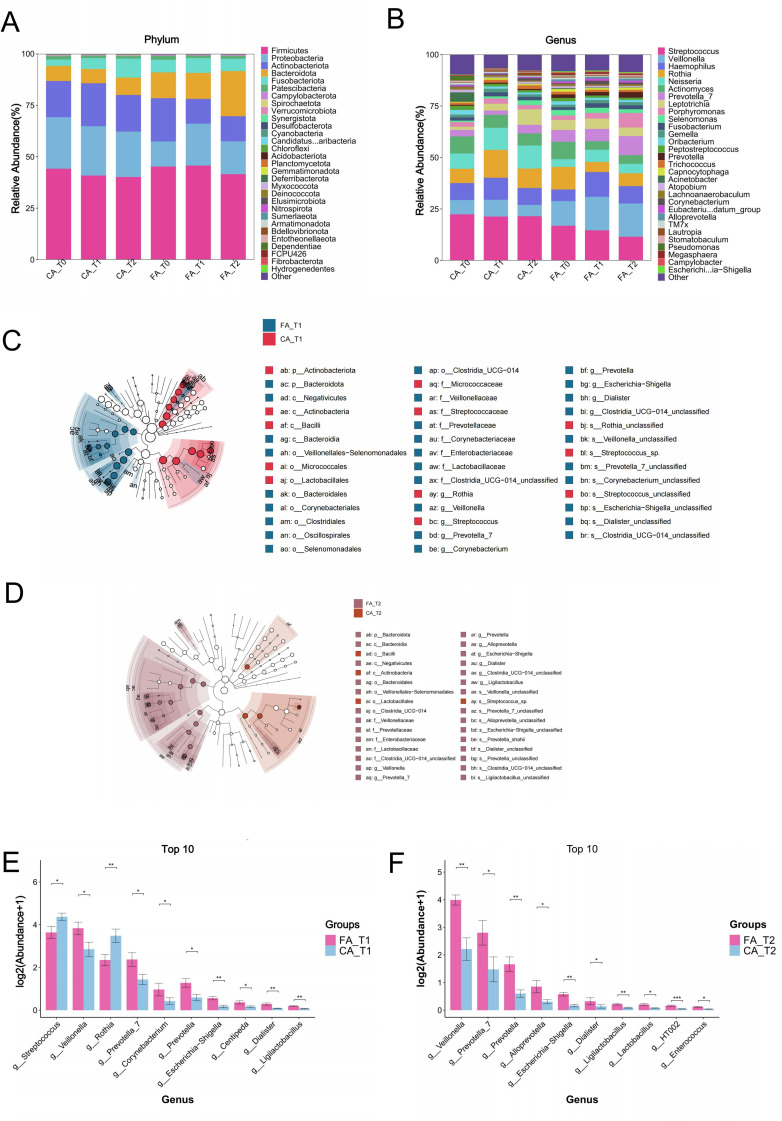
Microbial composition analysis of salivary samples from FA and CA groups. **(A)** Microbial community composition at the phylum level. **(B)** Microbial community composition at the genus level. **(C)** Phylogenetic tree of microbial communities at T1. **(D)** Phylogenetic tree of microbial communities at T2. **(E)** Differential microbial taxa between groups at T1. **(F)** Differential microbial taxa between groups at T2 (*P < 0.05, **P < 0.01, ***P < 0.001).

At the phylum level in saliva samples (**Figure 5A**), the dominant taxa in both FA and CA group were *Firmicutes*, *Proteobacteria*, *Actinobacteria*, *Bacteroidetes*, and *Fusobacteria*.

At the genus level in saliva samples (**Figure 5B**), the major taxa included *Streptococcus*, *Veillonella*, *Rothia*, and *Haemophilus*. The relative abundance of *Streptococcus* showed a progressive decline from T0 to T2 in FA group, whereas it remained stable in CA group. In contrast, *Veillonella* increased over time in FA group but exhibited little fluctuation in CA group.

LEfSe analysis, a statistical method identifying taxa with significantly differences (LDA > 3), revealed multiple discriminatory taxa between groups (**Figures 5C, D**). At T1, *Veillonella, Corynebacterium*, and *Prevotella* were significantly enriched in FA group, while *Rothia* was enriched in CA group. At T2, enrichment of *Veillonella, Prevotella*, and *Lactobacillus* was observed in FA group.

Quantitative assessment of these taxa (**Figures 5E, F**) further confirmed that FA group had significantly higher relative abundances of *Veillonella, Corynebacterium, Prevotella*, and *Lactobacillus* at both T1 and T2 compared with CA group (*P* < 0.05). Conversely, *Rothia* was consistently more abundant in CA group than FA group (*P* < 0.05).

**Figure 6:**
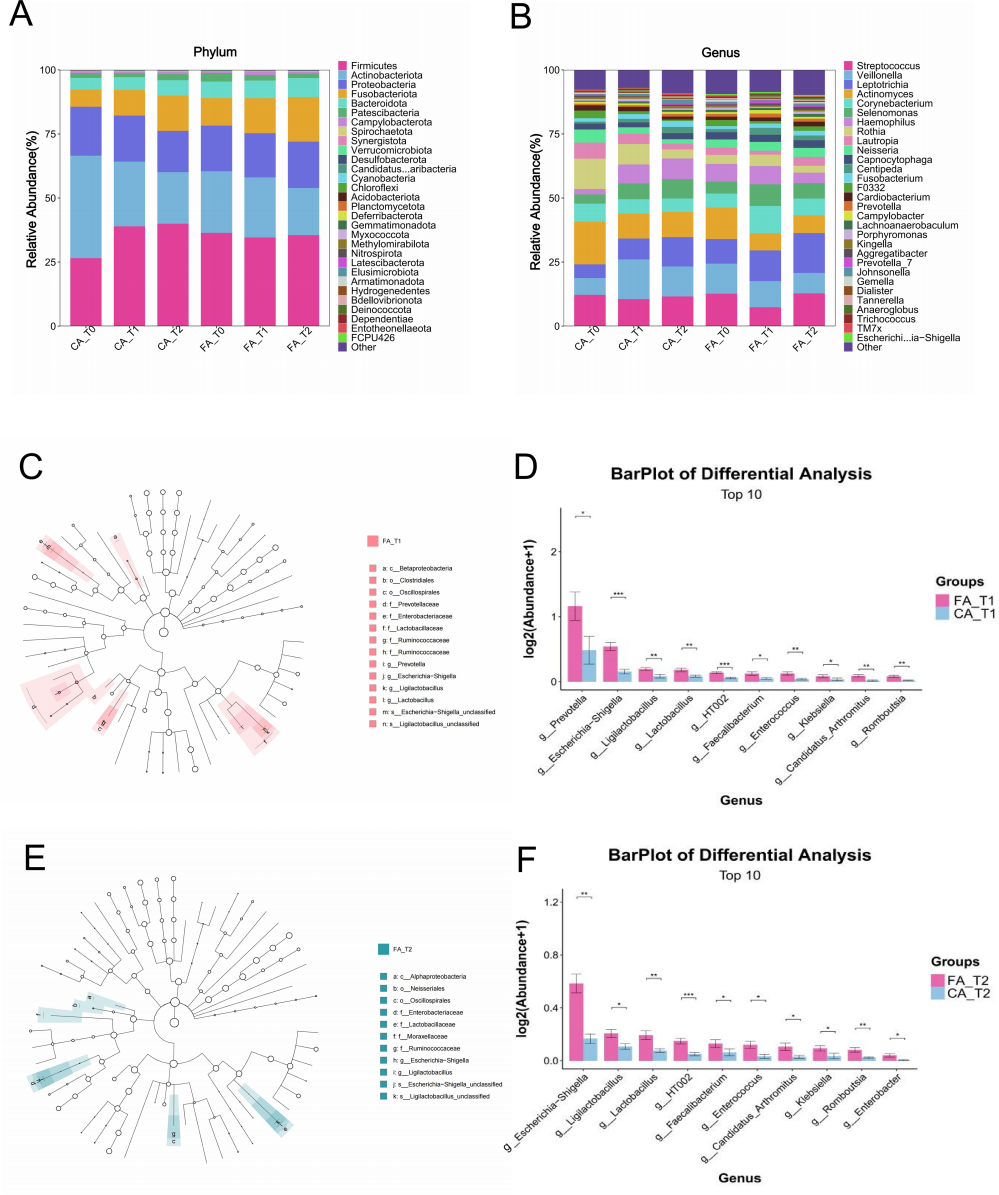
Microbial analysis of supragingival plaque samples from FA and CA group. **(A)** Microbial community composition at the phylum level. **(B)** Microbial community composition at the genus level. **(C)** Phylogenetic tree of bacterial communities at T1. **(D)** Differential taxa identified at T1. **(E)** Phylogenetic tree of bacterial communities at T2. **(F)** Differential taxa identified at T2 (*P < 0.05, **P < 0.01, ***P < 0.001).

In supragingival plaque samples at the phylum level (**Figure 6A**), both FA and CA group were dominated by *Firmicutes*, *Proteobacteria*, *Actinobacteria*, *Bacteroidetes*, and *Fusobacteria*.

At the genus level (**Figure 6B**), the predominant genera were *Streptococcus*, *Veillonella*, *Leptotrichia*, *Actinomyces*, and *Corynebacterium*. The relative abundance of *Actinomyces* showed a decreasing trend from T0 to T2 in both groups. In CA group, *Veillonella* and *Leptotrichia* increased over time, while their abundances remained stable in FA group.

LEfSe analysis (LDA > 3) identified several discriminatory taxa between the two groups (**Figures 6C, E**). At T1, *Lactobacillus* and *Prevotella* were enriched in FA group, while at T2, *Lactobacillus* remained the dominant discriminatory genus distinguishing FA group from CA group.

Quantitative analysis of differential taxa (**Figures 6D, F**) further demonstrated that the relative abundance of *Prevotella* at T1 was significantly higher in FA group compared with CA group (*P* < 0.05). In addition, *Lactobacillus* was consistently more abundant in FA group than CA group at both T1 and T2 (*P* < 0.05).

**Figure 7:**
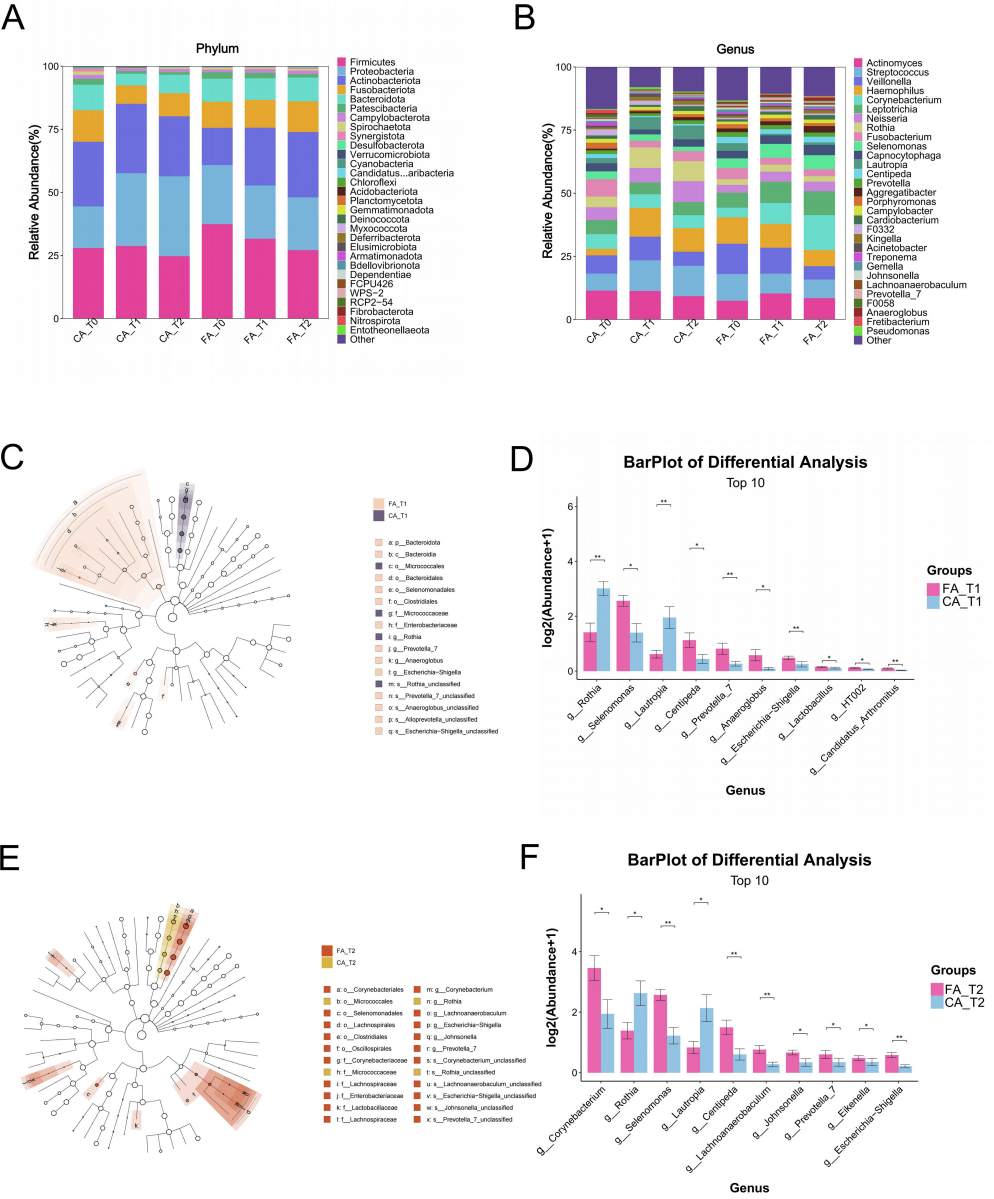
Microbial analysis of gingival crevicular fluid samples in FA and CA group. **(A)** Microbial community composition at the phylum level. **(B)** Microbial community composition at the genus level. **(C)** Phylogenetic tree of microbial communities between the two groups at T1. **(D)** Differential taxa were identified between the two groups at T1. **(E)** Phylogenetic tree of microbial communities between the two groups at T2. **(F)** Differential taxa were identified between the two groups at T2 (*P < 0.05, **P < 0.01, ***P < 0.001).

At the phylum level in GCF samples (**Figure 7A**), the dominant microbial communities in FA and CA group showed similar compositions, primarily including *Firmicutes*, *Proteobacteria*, *Actinobacteria*, *Bacteroidetes*, and *Fusobacteria*.

At the genus level (**Figure 7B**), the major taxa in both groups were *Actinomyces*, *Streptococcus*, *Veillonella*, *Haemophilus*, and *Corynebacterium*. The relative abundances of *Streptococcus* and *Haemophilus* decreased from T0 to T2 in FA group but increased in CA group, whereas *Corynebacterium* showed an increasing trend in FA group and remained stable in CA group.

LEfSe analysis (LDA score > 3) identified multiple discriminatory taxa between the two groups. At T1 (**Figure 7C**), FA group was enriched with *Selenomonas*, whereas CA group was enriched with *Rothia*. At T2 (**Figure 7E**), FA group was dominated by *Corynebacterium* and *Lactobacillus*, while CA group was dominated by *Rothia*.

Quantitative comparison of differential taxa further revealed that FA group had significantly higher relative abundances of *Lactobacillus* and *Selenomonas* than CA group at T1 (*P* < 0.05; **Figure 7D**). At T2, the relative abundances of *Corynebacterium* and *Selenomonas* were also significantly higher in FA group (*P* < 0.05; **Figure 7F**). By contrast, CA group exhibited significantly higher relative abundances of *Rothia* and *Lautropia* than FA group at both T1 and T2 (*P* < 0.05).

### 3.6 KEGG metabolic pathways analysis

**Figure 8:**
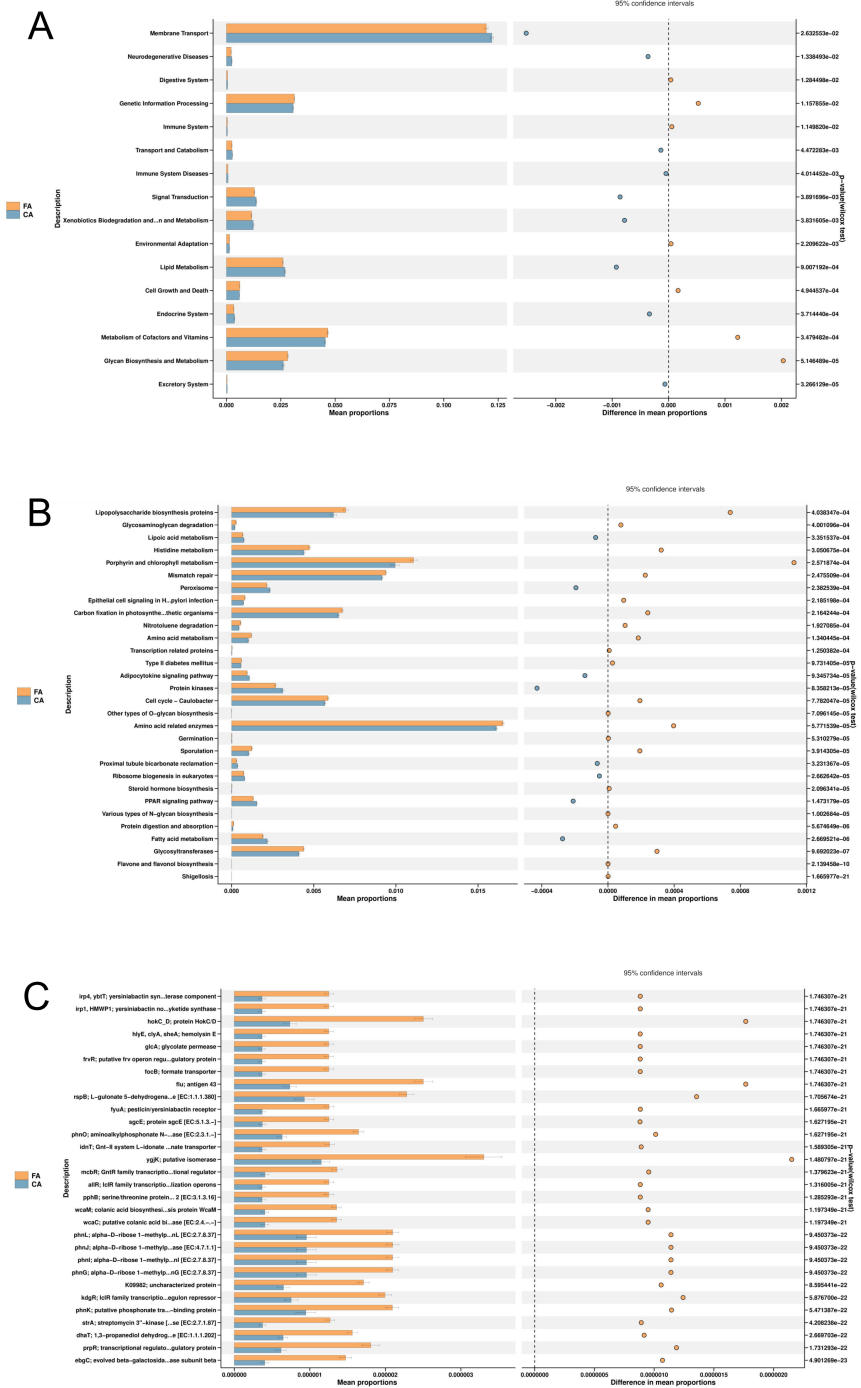
Differential KEGG metabolic pathway analysis between FA and CA groups. **(A)** KEGG Level 2 pathway differences; **(B)** KEGG Level 3 pathway differences; **(C)** KEGG Orthology (KO) pathway differences.

Functional prediction based on the KEGG database revealed distinct microbial metabolic potentials between the FA and CA group (**Figure 8**). In FA group, pathways related to carbohydrate metabolism were significantly enriched (*P* < 0.05), including glycan biosynthesis and metabolism, glycosaminoglycan degradation, and glycosyltransferases. Moreover, enrichment was also observed in the type II diabetes mellitus pathway and lipopolysaccharide biosynthesis proteins (*P* < 0.05). In contrast, CA group exhibited significant enrichment (*P* < 0.05) in pathways associated with lipid metabolism, such as the PPAR signaling pathway, adipocytokine signaling pathway, and fatty acid metabolism.

### 3.7 Correlation between 8-OHdG analysis (oxidative stress proxy), differential taxa and periodontal indices

**Figure 9:**
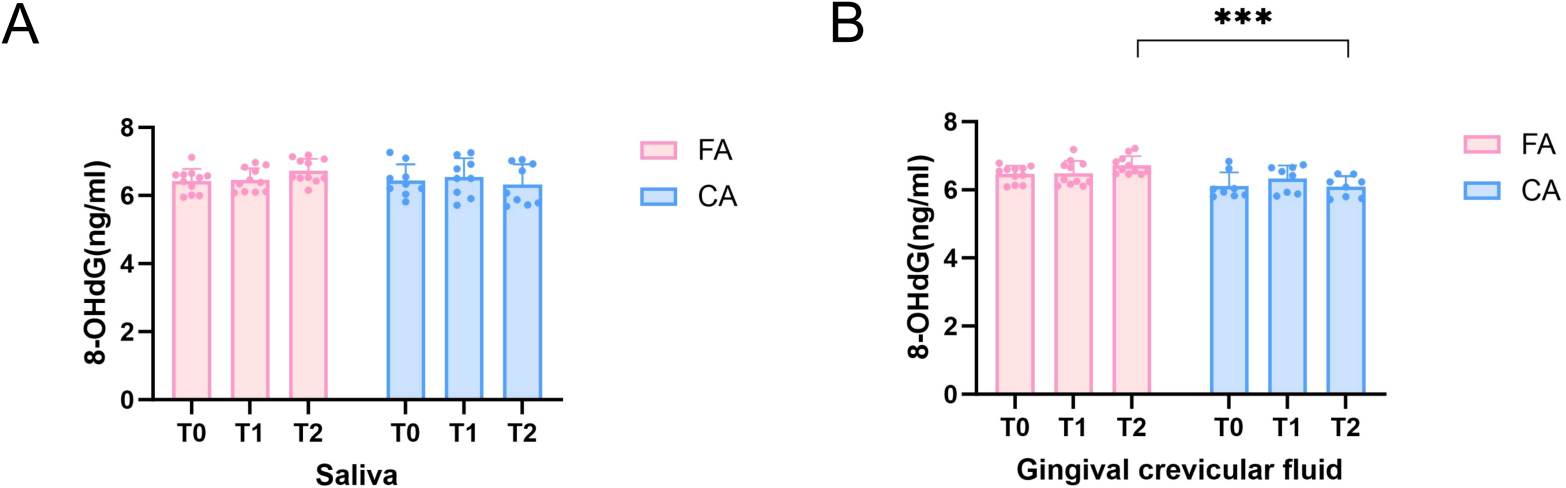
8-OHdG levels in saliva and GCF of FA and CA groups at T0, T1, and T2. **(A)** 8-OHdG levels in saliva; **(B)** 8-OHdG levels in GCF (*P < 0.05, **P < 0.01, ***P < 0.001).

8-OHdG was detected to indirectly reflect the ROS levels. The changes of 8-OHdG levels in salivary and GCF were presented in **Figure 9**. In saliva samples, there were no significant differences of 8-OHdG levels in either FA group or CA group at T0, T1 and T2 (**Figure 9A**). Similarly, in GCF samples, no significance differences of 8-OHdG levels in either FA group or CA group at T0, T1 and T2 were found (**Figure 9B**). However, in GCF samples, 8-OHdG levels in FA group were significantly higher than those in CA group at T2 (*P* < 0.001).

**Figure 10:**
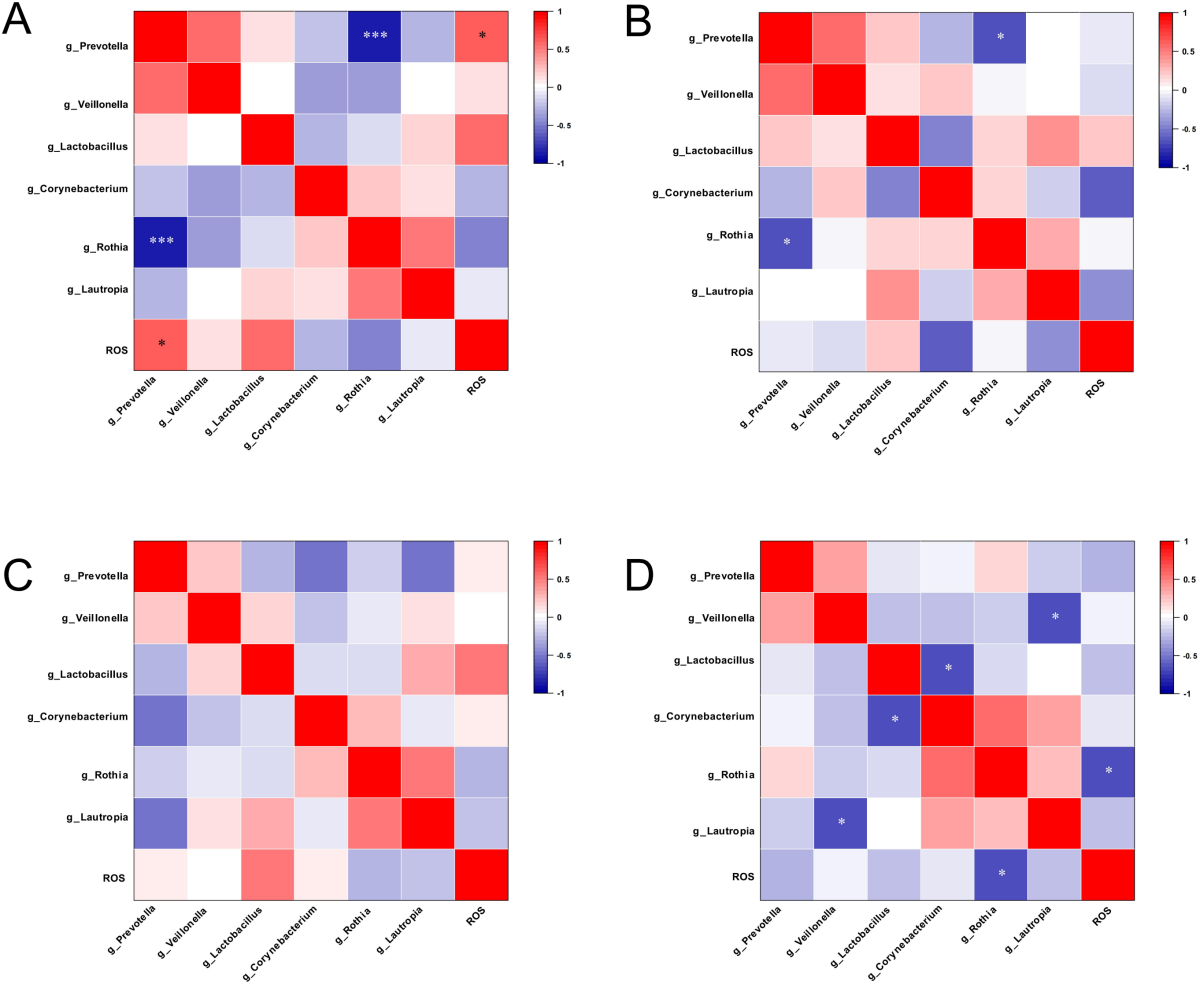
Correlation heatmaps between reactive oxygen species (ROS) and oral microbial taxa. **(A)** Correlations between ROS levels and microbial taxa in saliva from FA group; **(B)** Correlations between ROS levels and microbial taxa in saliva from CA group; **(C)** Correlations between ROS levels and microbial taxa in GCF from FA group; **(D)** Correlations between ROS levels and microbial taxa in GCF from CA group (*P < 0.05, **P < 0.01, ***P < 0.001).

We performed Spearman correlation analysis to evaluate the relationship between ROS levels and the differential taxa (**Figure 10**). In saliva samples, the relative abundance of *Prevotella* in FA group showed a significant positive correlation with ROS levels (*r* = 0.61, *P* < 0.05), whereas *Rothia* exhibited a strong negative correlation with *Prevotella* (*r* = -0.90, *P* < 0.001) (**Figure 10A**). In CA group, *Prevotella* was negatively correlated with *Rothia* (*r* = -0.67, *P* < 0.05) (**Figure 10B**). In GCF samples, significant correlations were also observed in CA group. As shown in **Figure 10D**, the relative abundance of *Rothia* was negatively correlated with ROS levels (*r* = -0.65, *P* < 0.05), *Veillonella* was negatively correlated with *Lautropia* (*r* = -0.70, *P* < 0.05), and *Lactobacillus* was negatively correlated with *Corynebacterium* (*r* = -0.68, *P* < 0.05).

**Figure 11:**
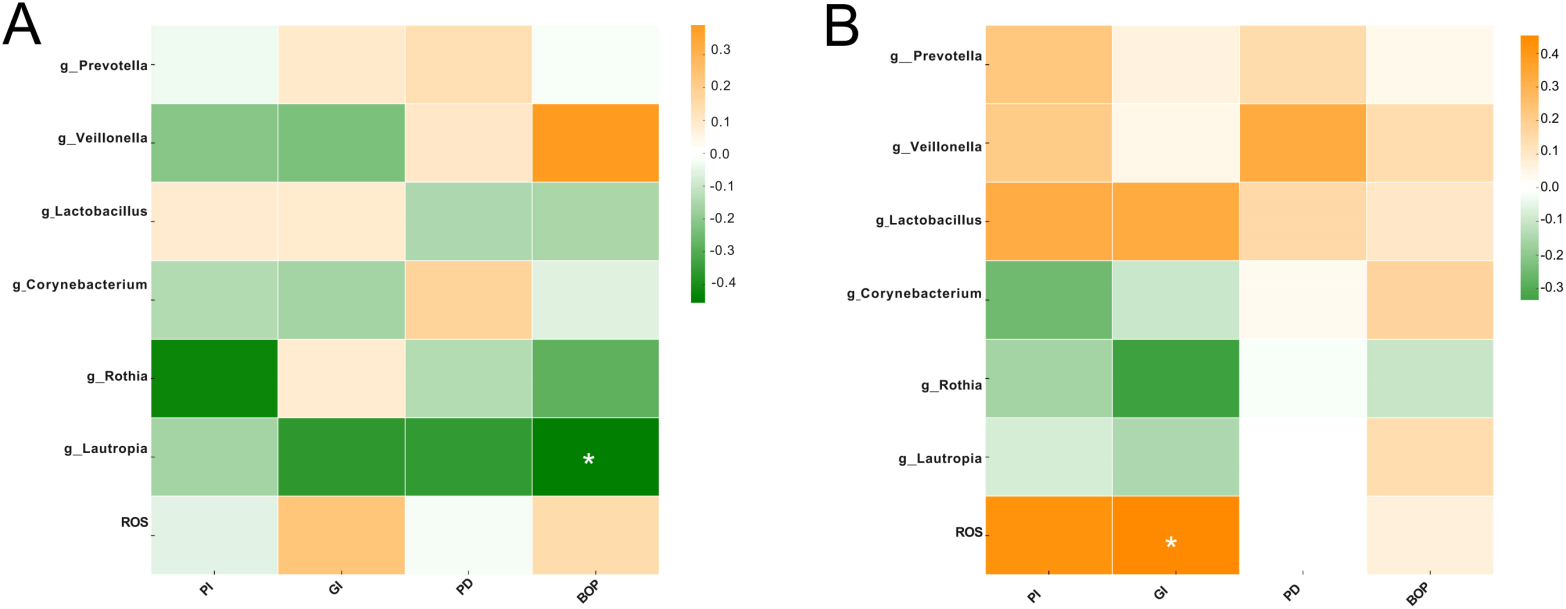
Correlation heatmap of periodontal clinical indices with ROS and oral microbial taxa. **(A)** Correlations between periodontal indices, ROS levels, and microbial taxa in FA group. **(B)** Correlations between periodontal indices, ROS levels, and microbial taxa in CA group (*P < 0.05, **P < 0.01, ***P < 0.001).

Correlation analysis revealed distinct associations between clinical periodontal indices, ROS levels, and differential microbial taxa (**Figure 11**). In FA group, BOP showed a significantly negative correlation with the relative abundance of *Lautropia* (**Figure 11A**). In CA group, GI showed a significantly positive correlation with ROS levels (**Figure 11B**).

## 4 Discussion

This study comprehensively evaluated the potential impact of clear aligners and fixed appliances on the oral micro environmental and periodontal health by analyzing saliva, supragingival plaque, and GCF samples collected at T0, T1, and T2. High-throughput 16S rDNA sequencing was integrated with clinical periodontal indices (PI, GI, BOP, PD) and ROS detection. The findings revealed differences between the two orthodontic modalities in maintaining of periodontal health, microbial community composition and functional potential, and oxidative stress levels.

The present study observed a progressive increase in PI, GI, PD and BOP in patients treated with fixed appliances as treatment duration extended. Conversely, patients in the CA group exhibited only minor fluctuations in PI and PD (**Figure 2**). These results demonstrated that clear aligners might provide potential advantage over fixed appliances in limiting plaque accumulation and reducing gingival inflammation. At T1 and T2, PI was significantly higher in FA group compared with CA group (**Figure 2**). These differences were likely attributable to the inherent design of the two orthodontic systems. The brackets, archwires, and bands of fixed appliances generate multiple plaque-retentive sites that were relatively difficult to clean thoroughly. On the contrary, the removability of clear aligners facilitated effective oral hygiene practices and might lowered the burden of plaque control (Kozak et al., 2021; Manphibool et al., 2023; Rouzi et al., 2023). As a sensitive indicator of gingival inflammation, BOP was used for early gingivitis detection and patient monitoring. Our results suggested that clear aligners might alleviate gingival inflammation. Moreover, our findings were also consistent with those reported by Chhibber et al, further supporting the potential positive role of clear aligners in maintaining periodontal health during orthodontic therapy (Chhibber et al., 2018).

To comprehensively evaluate changes in oral microecology, three representative sample types were selected: saliva, supragingival plaque, and GCF. Saliva offers a comprehensive representation of the diversity and abundance of oral microorganisms and has been widely recognized as a reliable tool for assessing the risk of caries and periodontal diseases (Haririan et al., 2014; Pedersen et al., 2018; Garcia et al., 2024). Supragingival plaque reflects the diversity and quantity of cariogenic bacteria, which are critical in the development of caries (Qudeimat et al., 2021), while GCF serves as an indicator of gingival and periodontal inflammatory status (Bibi et al., 2021; Yang et al., 2021). In this study, microbial communities of saliva, supragingival plaque, and GCF were profiled using 16S rDNA high-throughput sequencing for comprehensive and sensitive assessment (Shen et al., 2022).

Alpha diversity analysis reflects species richness and evenness within individual samples. Our study showed that, no significant differences were found in Chao1, ACE, Shannon, Simpson indices between CA and FA groups or among different time points in each group. These results were align with those of Liu et al (Liu et al., 2024), indicating that during the early treatment phase, neither appliance type significantly affected the overall richness and evenness of the oral microbiome. Beta diversity analysis assesses inter-sample community dissimilarities. Interestingly, our results demonstrated an obvious relatively separation of microbial community structures between CA and FA groups in saliva, supragingival plaque, and GCF (**Figure 4**). These findings suggested that while the “quantity” of microbial diversity remained largely stable, the “quality” of microbial community composition was to some extent influenced by different types of orthodontic appliances. The divergence might be attributed to two main factors. First, the surface characteristics of fixed appliances or clear aligners played an important role (Low et al., 2011; Kozak et al., 2021). The rough surfaces of fixed brackets and bands were prone to promote biofilm retention. Similarly, the long-term wear of clear aligners could lead to micro-abrasions and fissures on the surfaces of aligners which also favorable for the colonization of the plaque. Second, distinct impacts on the oral microenvironment, including variations in local oxygen tension and pH, contribute to this divergence. Notably, previous research had shown that as early as 48 hours after clear aligners placement, specific bacterial taxa (e.g., *Streptococcus* and *Corynebacterium*) could be detected colonizing on clear aligners’ surfaces (Low et al., 2011).

At the phylum level, *Firmicutes*, *Actinobacteria*, *Proteobacteria*, *Fusobacteria*, and *Bacteroidetes* were consistently identified as dominant taxa in both groups. Within *Firmicutes*, several genera were closely associated with oral diseases, including *Streptococcus*, *Lactobacillus*, and *Veillonella*. Among them, *Streptococcus mutans* which were highly acidogenic and aciduric, facilitated biofilm formation, and contributed to enamel demineralization (Shiotsu-Ogura et al., 2019). Whereas, *Streptococcus sanguinis* functioned as an early colonizer which might be potentially involved in plaque initiation. *Actinobacteria* also constituted a critical component of the oral microbiome, with *Actinomyces* sp*ecies* frequently linked to various oral pathologies. *Fusobacteria* were another key group, exemplified by *Fusobacterium nucleatum*, which played an important role in periodontal disease, caries, oral infections, and systemic disorders. Likewise, *Bacteroidetes* also had a huge impact on oral health and disease. For instance, genera such as *Porphyromonas*, *Prevotella*, and *Tannerella* were strongly implicated in periodontal disease, caries, and overall health condition (Yang et al., 2025).

Genus-level analysis showed that *Prevotella* was significantly more abundant in saliva and supragingival plaque samples from FA group than CA group. Previous studies had identified *Prevotella* as a key periodontal pathogen that produced diverse virulence factors capable of directly damaging periodontal tissues or inducing host immune responses, thereby driving periodontitis (Jiang et al., 2014; Sharma et al., 2022). Among its species, *Prevotella intermedia* had been reported to co-aggregate with *Porphyromonas gingivalis* and *Fusobacterium nucleatum* to form subgingival biofilms, enhancing pathogenicity and accelerating disease progression (Das et al., 2023). The present findings suggested that fixed appliances might be associated with the colonization of *Prevotella*, thereby increasing the potential risk of periodontitis. These results were consistent with those of Zheng et al., who also noted elevated *Prevotella* abundance in patients treated with fixed appliances (Zheng et al., 2024). Furthermore, the positive correlation between *Prevotella* and PI was reported previously, which supported the higher PI in FA group (Xie et al., 2025). This indicated that fixed appliances might facilitate plaque accumulation, with *Prevotella* contributing to biofilm formation and heightened inflammation risk.

In saliva, the FA group also showed significantly greater abundance of *Veillonella* at both T1 and T2 compared with CA group. Elevated *Veillonella* levels had been reported in periodontitis patients, where their metabolic products could impair periodontal tissues and trigger inflammatory responses (Ortiz et al., 2022). The current findings indicated that fixed appliances might be linked to *Veillonella* proliferation, thereby increasing susceptibility to periodontal inflammation. This observation was consistent with the findings of Shokeen Bhumika et al. who reported that fixed appliances might increase the risk of periodontal inflammation (Shokeen et al., 2022).

The present study demonstrated that the relative abundance of *Lactobacillus* was significantly higher in saliva, supragingival plaque, and GCF samples from the FA group compared with the CA group. Previous studies had shown that *Lactobacillus*, characterized by its acidogenic and aciduric properties, acted as a major cariogenic bacterium involved in the initiation and progression of dental caries (Gondo et al., 2024). Elevated levels of *Lactobacillus* had consistently been observed in saliva and supragingival plaque of caries patients compared with healthy individuals, supporting the notion that fixed appliances might facilitate its proliferation and thereby increase the risk of caries.

Similarly, *Corynebacterium* was significantly enriched in saliva and GCF samples from the FA group relative to the CA group. Evidence indicated a strong association between *Corynebacterium* and the onset of dental caries, highlighting its potential role in disease initiation (Jiang et al., 2014). The present findings therefore suggested that fixed appliances were easier to create an oral environment conducive to the growth of cariogenic bacteria, ultimately increasing caries susceptibility. This interpretation was consistent with the results of Wang and Shokeen Bhumika et al., further reinforcing the conclusion that fixed appliances might pose more risks for caries development (Wang et al., 2019; Shokeen et al., 2022).

Moreover, at both T1 and T2, the relative abundance of *Rothia* and *Lautropia* in GCF was significantly higher in CA group compared with FA group. Previous studies had demonstrated that *Rothia* contributed to oral health through its immunomodulatory and metabolic functions, with its abundance closely associated with a healthy oral status (Liu et al., 2023; Lundtorp Olsen et al., 2023). Similarly, *Lautropia* was frequently detected in healthy individuals and is regarded as a beneficial genus linked to oral homeostasis (Liu et al., 2023). The present findings therefore indicated that clear aligners were associated with the colonization of health-related taxa such as *Rothia* and *Lautropia*. These results were consistent with those reported by Zheng and Shokeen et al., supporting the notion that clear aligners might offer some unique advantages in maintaining oral health during orthodontic treatment (Shokeen et al., 2022; Zheng et al., 2024).

Functional prediction using PICRUSt2 (KEGG database) revealed distinct microbial metabolic potentials between the FA and CA groups (**Figure 8**). The FA group exhibited significant enrichment in carbohydrate metabolism–related pathways, including glycan biosynthesis and metabolism, glycosaminoglycan degradation, and glycosyltransferases. Carbohydrate metabolism was a central energy acquisition pathway for microbial growth, and its heightened activity is typically associated with enhanced microbial proliferation (Guo et al., 2025). Moreover, the FA group showed significant enrichment in the Type II diabetes mellitus pathway and in proteins involved in lipopolysaccharide (LPS) biosynthesis. LPS, the major structural component of Gram-negative bacterial cell walls, was a potent endotoxin and pro-inflammatory mediator (Hicks and Jia, 2018). The FA group showed increased abundance of Gram-negative pathogens such as *Prevotella*, along with higher periodontal inflammation indices and elevated ROS levels. These observations were associated with enrichment in carbohydrate metabolism-related pathways dependent microbes, including Gram-negative pathogens, and LPS biosynthesis proteins. The FA group exhibited enrichment in pathways potentially linked to local inflammatory responses and oxidative stress, which was consistent with the compromised periodontal status. It is important to note that PICRUSt2 inferences represent potential functions, not measured activities. The predicted enrichment of the Type II diabetes mellitus pathway based on metagenomic functional prediction merely reflected the differences in the potential function of the microbial community rather than direct changes in host systemic glucose metabolism. The clinical significance of this finding required further validation and investigation through integration with host-derived metabolic indicators.

In contrast, the CA group showed significant enrichment of lipid metabolism-related pathways, including lipid metabolism, adipocytokine signaling, and fatty acid metabolism. Lipids and their metabolites played essential roles in maintaining cell membrane stability, mediating signal transduction (including inflammatory signaling), and regulating energy storage (Parsons and Rock, 2013; VanHook, 2020). Previous studies suggested that specific lipid metabolic activities could modulate inflammatory responses and alleviate oxidative stress (Andersen, 2022). In this context, clear aligners influenced the microbiota by enhancing lipid metabolic activity, which showed enrichments in pathways associated with lipid metabolism. These functional features might be associated with the relatively stable periodontal condition and reduced ROS levels observed in the CA group.

ROS is a kind of oxygen-containing molecules or free radicals with high reactivity. Most ROS exist in the organism for a very short time, and it will quickly react with other molecules or be scavenged by the antioxidant system (Dumitru et al., 2025). Previous studies have identified that the stable metabolites of ROS mainly include lipid peroxidation products (Malondialdehyde, 4-Hydroxynonenal), DNA oxidative damage markers (8-OHdG) and oxidized glutathione. Among these, 8-OHdG is the most stable product of oxidative DNA damage caused by ROS. Thus, in our study, 8-OHdG was detected to indirectly reflect ROS levels with ELISA kit based on highly sensitive double-antibody sandwich method. Two specific antibodies bind to different epitopes of the target antigen, forming an “antibody-antigen-antibody” sandwich complex. This enables highly sensitive and specific quantitative or qualitative detection (Lu et al., 2025). Given the short half-life and low concentrations of ROS in the oral environment, this technique offers a reliable and effective approach for accurate measurement.

Recent studies showed that long-term psychological stress can disrupt the redox balance, leading to excessive accumulation of ROS, which exceeds the antioxidant defense capacity of cells, triggering oxidative stress and subsequently damaging biological macromolecules such as DNA, RNA, proteins and lipids (Yu et al., 2024). Thus, in order to minimize the influence of psychological stress on ROS, we used the PSS-10 to control the confounding factors. Our results showed that there were no significant differences between FA group and CA group in T0, T1, and T2. Therefore, we considered that the ROS levels measured in this experiment were reliable. Our study demonstrated the temporal changes of ROS levels in saliva and GCF during the orthodontic treatment (**Figure 9**). In saliva, there were no significant differences of ROS levels in either FA group or CA group during treatment duration (T0, T1 and T3). Similarly, no significant differences of ROS levels in either FA group or CA group in GCF samples during treatment duration (T0, T1 and T3). Although there were no significant temporal changes of ROS levels in either saliva or GCF, the ROS levels in FA group were obviously higher than those in CA group at T2 with statistical significance (*P* < 0.001). As GCF originated directly from gingival tissues, it could be viewed as a sensitive marker of local periodontal inflammation and oxidative stress (Bibi et al., 2021). The pronounced elevation of ROS in GCF at T2 indicated that fixed appliances might exert greater oxidative stress on periodontal tissues compared to clear aligners in orthodontic treatment process. Specifically, several mechanisms might explain this effect. First, degradation of metallic components such as brackets and archwires in the FA group might release nickel and chromium ions into the oral cavity, catalyzing ROS production through Fenton-type reactions (Abedini et al., 2009; Mahmud et al., 2018). Second, increased plaque accumulation and gingival inflammation associated with fixed appliances, reflected by higher GI values, might activate neutrophils and other immune cells, leading to respiratory bursts which could release large amounts of ROS (Kanzaki et al., 2017). Third, alterations in microbial community structure, including enrichment of pathogenic genera (*Prevotella* and *Veillonella*), as well as enhanced metabolic activities such as elevated LPS production and carbohydrate metabolism, might also directly or indirectly stimulate ROS generation (Kang et al., 2019).

In saliva, ROS levels in FA group were significantly positive correlated with the relative abundance of *Prevotella* (r = 0.61, *P* < 0.05). Previous studies (Ibrahim et al., 2017) have shown that *Prevotella* and its metabolic products could stimulate immune cells to produce ROS through their pro-inflammatory effects. *Prevotella* abundance was strongly negatively correlated with *Rothia* abundance (r = -0.90, *P* < 0.001). As a potential beneficial genus, *Rothia* likely limited the proliferation of pathogenic bacteria such as *Prevotella* through competitive interactions or inhibitory substances (Lundtorp Olsen et al., 2023). These associations suggested that the salivary microenvironment in FA group might shift towards a pro-inflammatory and pro-oxidative state.

In GCF, ROS levels in CA group showed a significantly negative correlation with the relative abundance of *Rothia* (r = -0.65, *P* < 0.05). Considering the enrichment of *Rothia* observed in CA group, this finding indicated that *Rothia* or the microenvironment mediated by *Rothia* might exert antioxidant effects or mitigate oxidative stress, thereby providing a potential lead for developing microbiota-based antioxidant strategies.

A significant positive correlation was identified between GI and ROS levels in the FA group (**Figure 11B**). This observation aligned with the classical model in which plaque biofilms, reflected by increased GI, stimulated gingival tissues and elicited inflammatory responses. Activated neutrophils infiltrated the tissues and released large amounts of ROS. Excessive ROS subsequently caused periodontal damage such as protein and DNA oxidation and fibroblast apoptosis, thereby perpetuating a vicious cycle (Tamaki et al., 2009; Kanzaki et al., 2017). These findings further integrated the microbial alterations observed in FA group (increased pathogenic taxa) with the deterioration of periodontal parameters (elevated GI) and the enhancement of oxidative stress (increased ROS).

## 5 Limitations and future directions

Several limitations should be noted. Notably, predicted functions from PICRUSt2 represent potential rather than actual microbial metabolic activity. First, the relatively small sample size (n = 12 per group; statistical power > 0.8; PASS 2021 software) largely dictated by practical and economic constraints might have reduced statistical power and limited the detection of subtle differences. Second, microbial functional analysis was based on 16S rDNA sequencing-derived predictions (PICRUSt2). These predictions represent potential functional capabilities of the microbial communities rather than direct measurements of microbial activity. While this method provides insights into functional potential, its accuracy is relatively lower than that of shotgun metagenomic or metatranscriptomic sequencing. Third, the associations observed between specific taxa and ROS levels were primarily statistical and lacked direct *in vitro* or *in vivo* validation to establish causality or clarify mechanistic pathways.

To overcome these limitations, future research should address these issues by increasing sample size and conducting multicenter studies to enhance generalizability. Advanced techniques such as metagenomic or metatranscriptomic sequencing could offer higher-resolution insights into microbial functional activities. Direct experimental validation in biofilm or animal models would help clarify the causal roles and molecular mechanisms of key taxa, such as *Prevotella* and *Rothia*, in ROS generation, inflammation, and periodontal tissue damage. Moreover, investigating microbiome-targeted interventions (e.g., probiotics or prebiotics) or antioxidant strategies may provide novel approaches to alleviate orthodontic treatment-related periodontal complications, particularly in patients with fixed appliances. Such studies will deepen our understanding of how different orthodontic modalities influence oral health and will provide stronger evidence to optimize periodontal management during orthodontic treatment.

## 6 Conclusion

This study examined the impact of clear aligners and fixed appliances on the oral microbiota of orthodontic patients. It also explored the relationship between microbial composition and ROS levels. Fixed appliances were associated with a higher relative abundance of periodontal and cariogenic pathogens, indicating a potential increased risk of periodontitis and caries. In contrast, clear aligners were linked to greater enrichment of beneficial taxa and observed alongside lower ROS levels in GCF, reflected by more favorable ROS indices. These findings suggest that clear aligners may provide potential advantages in preserving microbial homeostasis and reducing oxidative stress-related damage during orthodontic treatment. Additionally, correlation analysis identified *Prevotella* (a periodontal pathogen) and *Rothia* (a potentially beneficial genus) as key taxa likely involved in ROS modulation based on correlation analysis. These insights highlight promising research for monitoring oral health in orthodontic patients, preventing periodontal and caries-related complications, and guiding the development of novel oral materials with dual antimicrobial and antioxidant functions.

## Data Availability

The datasets presented in this study can be found in online repositories. The names of the repository/repositories and accession number(s) can be found below: NCBI, under BioProject PRJNA1355306 (https://www.ncbi.nlm.nih.gov/sra/PRJNA1355306).
