# Supplementary material for: Comparison of the effects of clear aligners and fixed appliances on the oral microbiota and reactive oxygen species: a prospective study
Source: Front Cell Infect Microbiol. 2026 Jan 23;15:1738047. doi: 10.3389/fcimb.2025.1738047 (PMC12876197; doi:10.3389/fcimb.2025.1738047)
Supplement: Supplementary file 1 [file DataSheet1.pdf]

Supplementary Table 1 questionnaire

| ID | Question/value |
|---|---|
| 1 | Name |
| 2 | Height |
| 3 | Weight |
| 4 | Have you been diagnosed with any systemic diseases (such as blood, cardiovascular, respiratory, digestive, immune system,or neurologic disorders) in the past month?[Yes/No] |
| 5 | Have you been diagnosed with infectious diseases in the past month?[Yes/No] |
| 6 | Have you experienced any allergic reactions in the past month?[Yes/No] |
| 7 | Have you experienced recurrent aphthous ulcer in the past month?[Yes/No] |
| 8 | Did you have diarrhea last month?[Yes/No] |
| 9 | Did you have a sleep disorder last month? [Yes/No] |
| 10 | Did you have dental treatments (such as fillings, extraction, or dental implants)?[Yes/No] |
| 11 | Did you taken antibiotics last month?[Yes/No] |
| 12 | Did you taken antioxidant supplements last month?[Yes/No] |
| 13 | Did you eat any desserts(such as cakes, candies) last month? [Yes/No] |
| 13.1 | Frequency per week |
| 14 | Did you drink sugar-sweetened beverage last month? [Yes/No] |
| 14.1 | Frequency per week |
| 15 | Did you drink coffee last month? [Yes/No] |
| 15.1 | Frequency per week |
| 16 | Did you drink alcohol last month? [Yes/No] |
| 17 | Do you brush your teeth three times a day? [Yes/No] |
| 18 | Do you use toothpaste with fluoride?[Yes/No] |
| 19 | Do you use dental floss? [Yes/No] |
| 20 | Do you wear your clear aligners at least 22 hours per day? [Yes/No] |

Supplementary Table 2 PSS-10

| Item | FA | | | CA | | |
|---|---|---|---|---|---|---|
| | T0 | T1 | T2 | T0 | T1 | T2 |
| Q1 felt anxious about something that happened unexpectedly? | 1.42±0.67 | 1.58±0.67 | 1.50±0.67 | 1.25±0.62 | 1.42±0.51 | 1.33±0.49 |
| Q2 felt unable to control the important things in your life? | 1.50±0.52 | 1.67±0.49 | 1.67±0.49 | 1.42±0.67 | 1.42±0.67 | 1.50±0.52 |
| Q3 felt nervous and stressed? | 1.42±0.51 | 1.42±0.51 | 1.42±0.51 | 1.42±0.51 | 1.33±0.49 | 1.33±0.49 |
| Q6 found that you could not cope with all the things that you had to do? | 1.58±0.67 | 1.58±0.51 | 1.67±0.49 | 1.58±0.67 | 1.58±0.51 | 1.75±0.45 |
| Q9 been angered because of things that were outside of your control? | 1.67±0.39 | 1.42±0.51 | 1.50±0.52 | 1.42±0.51 | 1.42±0.51 | 1.5±0.52 |
| Q10 felt difficulties were piling up so high that you could not overcome them? | 1.67±0.65 | 1.67±0.65 | 1.75±0.45 | 1.75±0.45 | 1.75±0.45 | 1.75±0.45 |
| Q4 felt confident about your ability to handle your personal | 2.25±0.75 | 2.17±0.83 | 2.25±0.75 | 2.25±0.45 | 2.33±0.49 | 2.17±0.39 |

| | | | | | | |
|---|---|---|---|---|---|---|
| problems? | | | | | | |
| Q5 felt that things were going your way? | 2.50±0.67 | 2.17±0.72 | 2.08±0.67 | 2.42±0.67 | 2.33±0.65 | 2.33±0.65 |
| Q7 been able to control irritations in your life? | 2.33±0.78 | 2.50±0.67 | 2.25±0.62 | 2.33±0.49 | 2.50±0.52 | 2.42±0.51 |
| Q8 felt that you were on top of things? | 2.50±0.52 | 2.33±0.49 | 2.33±0.49 | 2.58±0.51 | 2.58±0.51 | 2.67±0.49 |
| Total | 18.3±2.77 | 18.5±2.5 | 18.4±2.68 | 18.4±2.19 | 18.6±1.78 | 18.7±1.76 |

**P*<0.05, ***P*<0.01, ****P*<0.001

## Supplementary Figures

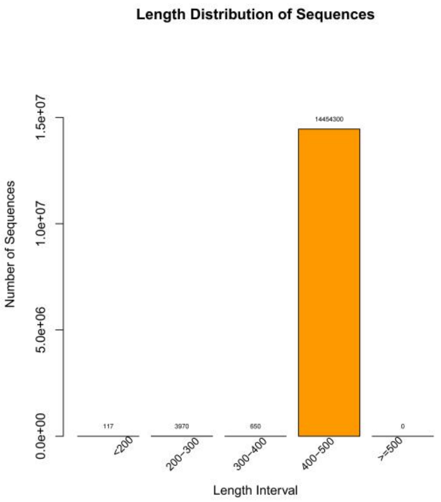


**Supplementary FIGURE 1.** Length of microbial sample sequence

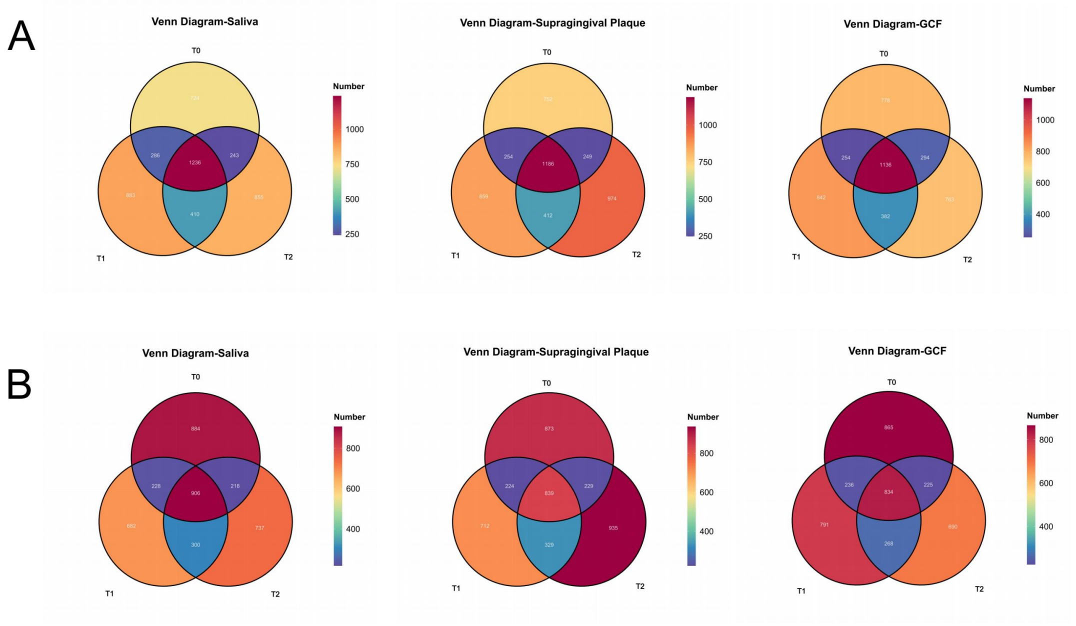


**Supplementary FIGURE 2.** Venn diagram analysis of microbial species in saliva, supragingival plaque, and gingival crevicular fluid samples from the FA and CA groups at T0, T1, and T2
